# Algorithmic Entropy and Landauer’s Principle Link Microscopic System Behaviour to the Thermodynamic Entropy

**DOI:** 10.3390/e20100798

**Published:** 2018-10-17

**Authors:** Sean Devine

**Affiliations:** School of Management, Victoria University of Wellington, P.O. Box 600, Wellington 6140, New Zealand; sean.devine@vuw.ac.nz

**Keywords:** algorithmic information theory, algorithmic entropy, non-equilibrium thermodynamics, distance from equilibrium, Landauer’s principle, conservation of bits

## Abstract

Algorithmic information theory in conjunction with Landauer’s principle can quantify the cost of maintaining a reversible real-world computational system distant from equilibrium. As computational bits are conserved in an isolated reversible system, bit flows can be used to track the way a highly improbable configuration trends toward a highly probable equilibrium configuration. In an isolated reversible system, all microstates within a thermodynamic macrostate have the same algorithmic entropy. However, from a thermodynamic perspective, when these bits primarily specify stored energy states, corresponding to a fluctuation from the most probable set of states, they represent “potential entropy”. However, these bits become “realised entropy” when, under the second law of thermodynamics, they become bits specifying the momentum degrees of freedom. The distance of a fluctuation from equilibrium is identified as the number of computational bits that move from stored energy states to momentum states to define a highly probable or typical equilibrium state. When reversibility applies, from Landauer’s principle, it costs $k_{B}ln2T$ Joules to move a bit within the system from stored energy states to the momentum states.

## 1. Introduction

Mathematicians have developed algorithmic information theory (AIT), and the associated concept of Kolmogorov or algorithmic complexity, to measure the computational resources needed to describe an object in terms of the length of the shortest string of characters that represent the object. In the mathematical arena, AIT has been used to define a robust measure of randomness [1,2], and to provide deeper insights into Gödel’s theorem [3]. The approach has also been applied to modelling data with the ideal form of the minimum description level [4]. It provides a universal similarity metric to categorise biological sequences [5,6] and for Bayesian prediction [7]. Others have used AIT to inquire into deep philosophical questions about the universe [8,9,10,11]. A comprehensive review can be found in Li and Vitányi [12].

In more recent papers, the algorithmic probability, which gives rise to the universal distribution, has been used to provide numerical estimates in a computational hierarchy [13], for analysing cognitive and behaviour data [14,15]. Additionally, the cost of reprogramming a generative mechanism has been illustrated by exploring networks using the maximum entropy principle [16], as has how evolving objects process information [17]. The algorithmic structure in natural environments has been explored by Zenil et al. [18]. Modelling mutations at the algorithmic level are explored in [19].

An important feature is that the algorithmic complexity, the length of the shortest self-delimiting algorithm that specifies the string representing a configuration in the natural world, is an entropy measure in bits that aligns in practical situations with the traditional entropies. For example, Devine [20] has used AIT to show there is no need to define a fourth law of thermodynamics to explain order in the universe, as has been postulated by the intelligent design community ([21]). More recently, algorithmic entropy has been used to track the emergence of order in far-from-equilibrium biological and economic systems [22,23].

This article shows how the mathematical tools of algorithmic information theory can be applied to the natural world in order to address the thermodynamic behaviour of systems, such as living systems, that need to be sustained distant from equilibrium. In particular, as the algorithmic entropy provides an entropy measure for both a non-equilibrium microstate and a typical equilibrium microstate, it can be used to explore the energy and entropy requirements of sustainability. Even so, the algorithmic entropy approach has not been widely used as a scientific tool because the basic framework needs to be extended. The following points provide a background to the article to explore the conceptual framework of AIT and how it can be applied effectively.

- The configuration of a natural system can be represented by a binary string in its state space, i.e., a string that specifies the instantaneous position, momentum, and stored energy states of all the species in the system. As natural laws can be understood as computations on a real-world universal Turing machine (UTM), the algorithmic entropy of a natural world configuration can be defined as the fewest computational bits that generate this string. As is discussed later, because a laboratory UTM can simulate the real-world UTM, the number of bits in the appropriately coded laboratory computer that halts after generating the configuration, specifies its algorithmic entropy to within a machine-dependent constant. As only entropy differences are of significance, the constant is irrelevant. The laboratory UTM that measures algorithmic entropy differences in the real-world is a measuring device that captures the algorithmic entropy of the natural system.
- The algorithmic entropy has always been recognised as a measure closely related to the Shannon entropy and the entropy of statistical mechanics (see [24,25]). However, once the specific requirements associated with real world reversible computations are properly accounted for, it is shown here that, allowing for units, the algorithmic entropy of an equilibrium or the typical microstate in an isolated system is identical to the thermodynamic entropy of the macrostate containing the set of allowable microstates. As shown in Section 3, because the algorithmic entropy of a typical macrostate is the same as the thermodynamic entropy, the algorithmic entropy can be used to quantify thermodynamic processes.
- The thermodynamic entropy of a thermodynamic macrostate in an isolated system is primarily determined by the most probable set of states. In contrast, the algorithmic entropy is conceptually different as it is the number of bits needed to specify an instantaneous microstate of the system. However, this is no different to recognising that the energy of a thermodynamic macrostate at an instant is actually the energy stored in the instantaneous microstate. Furthermore, in an isolated system, all microstates have the same algorithmic entropy, as they are connected by reversible processes, in which case the instantaneous configuration that is termed a “fluctuation from equilibrium” in an isolated system is here seen as a fluctuation from a typical or an equilibrium microstate that belongs to the most probable set of states.
- It is shown that, allowing for units, the number of bits specifying a microstate in the most probable set of states corresponds to the thermodynamic entropy of the isolated macrostate (see Section 3). For this reason, because the algorithmic entropy corresponds to the thermodynamic entropy when most of the bits specify momentum states, the algorithmic entropy can be termed the “realised entropy”. On the other hand, the bits specifying stored energy states are not usually seen as contributing to the thermodynamic entropy of the macrostate. As it is argued that bits, like energy, are conserved, the bits that specify a fluctuation from equilibrium mainly specify the stored energy or potential energy states. These bits can be termed “potential thermodynamic entropy” and only become realised when the energy associated with these bits diffuses through the system as heat, in which case bits specifying stored energy states become bits specifying momentum states.
- The distance from equilibrium of a fluctuation from a typical equilibrium state is the number of bits that shift from the stored energy states to the momentum states as the system trends to the most probable set of states. However, such a fluctuation from the most probable set of states in an isolated system is distinct from a system where the initial state is not just a fluctuation, but instead is a far-from-equilibrium configuration. In the latter case, when such a system trends to the most probable set of equilibrium states, the Boltzmann entropy increases as more states become available. In this different case, the distance from equilibrium is the number of bits that must enter the system for it to settle in an equilibrium configuration.
- The thermodynamic cost of maintaining a system distant from equilibrium can be understood in terms of compensating for the bit flows that attempt to drive the system to the most probable set of states. In the natural world, bits are computational instructions, embodied in the physical laws that specify the interactions between different real world states. As is discussed later in Section 6, Landauer’s principle [26] can be used to establish the thermodynamic cost of transferring bits out of a system and within a system [22,23,27] for a real-world reversible process. For a system to be stable in a homeostatic far-from-equilibrium set of configurations, both the energy flows into and out of the system, as well as the bit flows in and out, must balance. The paper explores the conceptual framework behind this principle to provide confidence in the manner in which the principle should be applied.
- In this paper, it can be seen that the algorithmic entropy, by focusing at the level of the microstate, provides a deterministic understanding of the manner in which the thermodynamic entropy increases as a system trends to equilibrium in terms of the computational processes involved. When ordering occurs, such as when magnetic spins align in a magnetic system, bits previously specifying random magnetic states become bits specifying momentum states raising the temperature. If the phase change in the spin system is to be locked in, bits from the momentum states must exist to lower the temperature. Bits can be tracked entering and leaving the system, but bits are conserved when isolated.

Mathematicians use the phrase “more complex” to describe a string representing a system that is more random, which, from both Shannon information theory and algorithmic points of view, embodies more information as bits. However, scientists tend to use “more complex” intuitively to mean a complicated or sophisticated system that shows a deep ordered structure, but which is anything but random. In order to avoid confusion, here the word “ordered” will be used to mean a low algorithmic entropy system that might be deemed to be complex in the intuitive sense, while the word “information” will be used in the mathematical sense to mean the number of bits needed to describe the system algorithmically.

## 2. Formal Outline of the Algorithmic Entropy

The basic concept of AIT was originally conceived by Solomonoff [28], while Kolmogorov [29] and Chaitin [30] independently formalised the approach. The approach is based on the recognition that highly ordered structures are simpler to describe. For example, the sequence formed by drawing 100 balls from a lotto urn must specify each of the 100 digits, whereas the first 100 digits of π can be generated by a simple algorithm. A natural system can be represented by a binary string of digits, denoted by *s*, that represents an instantaneous configuration of the system in an appropriate state space. If the string that describes a particular structure shows order, features or pattern, rather than specifying each character in the string separately, a shorter algorithmic description can be found to generate the string. The shortest description of a configuration in the natural world, such as a tree, is invariably given by the bits in the algorithm embodied in the DNA of the seed, coupled with the bits needed to specify the resources that the DNA instructions access to grow the tree.

The laws that shift one configuration to another in the natural world are computations on a real-world UTM. As one UTM can simulate another to within a constant, a real-world computation can in principle be simulated in detail on a laboratory reference UTM [12,31]. The length of p*, the shortest algorithm that generates the string on a reference UTM and then halts, is variously known as the “Kolmogorov complexity”, the “algorithmic complexity”, or the “programme sized complexity”.

The instructions in algorithms can be coded either with end markers to indicate when an instruction terminates or with self-delimiting coding that allows each instruction to be completed without the need for end markers. In the latter case, as no code can be a prefix of another all the codes must come from a prefix-free set [31,32,33] (see also [12]). In this case, the algorithm’s length defines the algorithmic entropy or the information content of the string. This coding increases the length of the algorithm specifying a string of length *N* by about $log_{2}N$. However, as real-world computational instructions are automatically self-delimiting, the length of the self-delimiting algorithm that specifies the string representing a natural system is an entropy measure that aligns with the Shannon and the thermodynamic entropy.

As is shown later in Section 4.2, because the algorithmic entropy is a state function, only algorithmic entropy differences have a physical meaning and differences are independent of the UTM used. Alternatively, one can choose an appropriate zero of algorithmic entropy [34] to eliminate computer dependence. In practice, a simple reference UTM is envisaged where most of the instructions occur as part the programme, ensuring that only a minimal set of logic gates (or their real-world equivalent) need to be part of the computational hardware.

### 2.1. Specifying the Shortest Algorithm

In formal terms, let U(p) be the computation using the self-delimiting programme *p* on the reference UTM denoted by *U*, and which halts when the output $s_{i}$ is generated. i.e., $U\left(p\right)=s_{i}$. Here, the vertical lines around *p*, as in |p|, represent the number of programme bits in the algorithm, in which case the algorithmic entropy or programme sized complexity is

$$H_{U}\left(s_{i}\right)=minimum\;\left|p\right|\;such\;that\;U\left(p\right)=s_{i}.$$

Here, and in what follows, the minimum programme *p* will be denoted by p*. Bennett [24] and Zurek [35] have shown that self-delimiting coding leads to the Kraft inequality, allowing subroutines to be joined to form larger algorithms without increasing the length of the description by more than a bit or two [36]. As only algorithmic entropy differences are of physical significance, in what follows, an appropriate algorithmic entropy zero is assumed to eliminate the machine dependence and any simulation constant. As this allows $H_{U}\left(s_{i}\right)$ to be machine-independent, the suffix *U* can be dropped and the algorithmic entropy becomes $H(s_{i})$. Conceptually, this implies that the number of bits needed to shift one state in the natural world to another is the same as that determined by a laboratory simulation of the natural process.

Like the Shannon entropy, the conditional algorithmic entropy can be defined when the outcome depends on information already given. In algorithmic terms, given an input string $s_{0}$, the conditional algorithmic entropy is the extra information in the shortest algorithm that calculates the output string $s_{i}$. Here $H\left(s_{i}|s_{0}\right)=\left|p^{*}\right|$ is the conditional algorithmic entropy term where p* is the shortest programme that shifts $s_{0}$ to $s_{i}$ by the computation $U\left(p^{*},s_{0}\right)=s_{i}$. Chaitin [31] shows,

(1) $$H\left(s_{i}\right)\leq H\left(s_{0}\right)+H\left(s_{i}|s_{0}^{*}\right).$$

The shortest description of $s_{0}$, namely $s_{0}^{*}$, is needed for consistency in the right-hand term of the equation. Generally, when $H(s_{i})$ is not completely dependent on string $s_{0}$, the ≤ sign is needed. However, in a reversible real-world computation, an equal sign is needed because $s_{0}$ must always be a precursor of $s_{i}$.

### 2.2. The Provisional Entropy

Laboratory measures such as temperature and pressure are used by the observer to characterise the macroscopic properties of a thermodynamic system even though they are dependent on its microscopic behaviour. This allows one to specify a particular microstate by an algorithm that specifies or generates the properties of the macrostate, coupled with an algorithm which, when given the macrostate properties, generates a particular microstate. The algorithmic entropy so obtained is called the provisional entropy [34]. The word provisional is used, as in some situations better knowledge might give rise to a shorter algorithm. The concept of provisional entropy is similar to Kolmogorov’s algorithmic minimum sufficient statistics (AMSS) [25] except that the AMSS approach always seeks the minimum set containing the string of interest, whereas the real-world macrostate must include all microstates consistent with the macrostate. As used in the natural world, the provisional entropy is the algorithmic entropy for each microstate in a set of microstates with a common structure. It is consistent with the generalised Gács’ Boltzmann entropy [12,37] and is a substitute for Zurek’s physical entropy [35].

However, as bits are conserved in a reversible natural system, the path to generating a particular real-world configuration invariably includes the specification of the macrostate.

More formally, the algorithm specifying a particular microstate in a thermodynamic macrostate has two parts. The first specifies the macrostate in terms of the properties of the set that contains the microstates. The macrostate properties are determined by parameters such as volume, energy, and number of particles. From these, the expectation values of temperature and pressure can be derived. The number of bits in the algorithm specifying the macrostate is represented by $H_{set}$. The second term identifies the specific string of interest given the properties of the macrostate. As a consequence, the provisional entropy $H_{prov}\left(s_{i}\right)$ of microstate $s_{i}$ is given by [34]:

$$H_{prov}\left(s_{i}\right)=H_{set}+H\left(s_{i}|set\right).$$

As the second term is the length of the algorithm to pick out string $s_{i}$, $H\left(s_{i}|set\right)=H_{Shannon}$, where $H_{Shannon}$ is the Shannon entropy of all microstates within the macrostate. The first term defining the macrostate is often small compared with the Shannon term, or for an isolated system can be taken as given. As a consequence, for a given thermodynamic macrostate, the number of bits needed to specify a particular microstate in a macrostate can be taken to be the Shannon entropy of all the microstates in the macrostate (see [34]).

## 3. The Algorithmic Entropy of a Real-World Thermodynamic Macrostate

An isolated thermodynamic system, which by definition exchanges neither energy nor matter with the environment, settles in a thermodynamic macrostate. Because here we are concerned with the exact specification of real-world states, and detailed computational paths, the phase or state space resolution, given by the volume of the smallest elementary cell, is taken to be $h^{3}$, the cube of Plank’s constant.

In this case, if there are Ω available microstates in the microcanonical ensemble, the Shannon entropy, $H_{Shannon}$, corresponds to the number of bits $N=log_{2}\Omega$ needed to identify a particular microstate. As mentioned in Section 2.2, given the macrostate, the algorithmic entropy of a particular microstate in the set defining the macrostate is also N bits, as this amount of bits is needed to specify the microstate in the set. Hence, for all microstates $H\left(s_{i}\right)=H_{Shannon}$. A connection can now be made between these two entropies and the Gibbs’s entropy. If the probability of a particular microstate in the macrostate is $p_{i}$, the Gibbs’ entropy is $S=-\Sigma_{i}p_{i}lnp_{i}$, over all the microstates. As for the isolated system, all Ω microstates are equally likely, the probability of a particular microstate is 1/Ω, and the Gibbs entropy becomes $S=k_{B}ln\Omega$, which is equivalent to $k_{B}ln2H_{Shannon}=k_{B}ln2H\left(s_{i}\right)$. Jaynes [38] has shown that the Gibbs entropy corresponds to the thermodynamic entropy. It follows that the algorithmic entropy for a set of equally likely microstates, when multiplied by $k_{B}ln2$, corresponds to the Gibbs’ entropy, allowing for units. As a consequence, the algorithmic entropy of a microstate in a macrostate of an isolated system is the same as the thermodynamic entropy, even though the algorithmic entropy is identified with a particular microstate. Just as the energy of a macrostate is actually embodied in the degrees of freedom of the microstate, so to is the algorithmic entropy. Section 6.3 shows that this is consistent with Landauer’s principle. When bits are reversibly transferred to the environment as heat, the energy flow is $k_{B}ln2T$ Joules per bit, showing how thermodynamic entropy flows are related to bit flows. It is only when these bit flows become irreversible does the overall entropy increase.

For an isolated system, the macroscopic of parameters such as pressure and temperature are average quantities that adjust as the configuration changes. From the perspective of an external observer, the observed parameters correspond to the expectation values, which primarily depend on the most probable set of states in the thermodynamic macrostate.

In what follows, to simplify discussion, rotational, vibrational, and translational states will be termed the momentum states, as it is these that embody kinetic energy and determine the temperature of the system, while other states, such as electronic states or chemical bonding states, will be identified as stored energy states. Furthermore, equilibrium refers to the most probable set or typical set of microstates. If the system does not exist in a highly probable state, the system rapidly trends toward such a state under the second law of thermodynamics.

## 4. Perspectives of Real-World Computations

The algorithmic entropy is a measurement tool calibrated in bits to allow a laboratory UTM to specify a real-world configuration. The real-world states and computations are not usually specified in binary notation but can always be converted into binary equivalents. As natural laws require a real-world computation to be reversible, a laboratory simulation cannot adequately account for all algorithmic properties in the natural world as outlined below.

- Instructions underpinning real-world computations are self-delimiting and are ongoing, only stopping when an external event impacts on the system. Ongoing computations in the natural world are parallel computations, where what might be deemed a subroutine by the observer continuously feeds its varying outputs into what would be termed a register. This register is regularly interrogated by the other routines until an appropriate input becomes available. As a consequence, real-world subroutines do not need to halt. It is the observer that requires subroutines to halt, so that the number of bits that characterise a microstate at an instant can be tracked.
  Section 4.2 argues that, for a reversible system, provided the bits are already in the system and the net flow of bits in an out are tracked, the algorithmic entropy obtained by tracking bits to the halt instant is the same as that obtained from the halting algorithm. This understanding provides insights into the computational requirements of maintaining a system far-from-equilibrium.
- A real-word computation enacts physical laws captured in the behaviour of atoms and molecules. These species act as reversible gates from a computational perspective, and the instructions embodied in the gates determine the computational trajectory. The behaviour of the gates is simulated on the laboratory reference computer by a programme. The programme is usually considered to be distinct from the string representing the bit settings of actual states. A difficulty arises, as reversibility, a critical characteristic of a real-world computation, is not usually built into a laboratory programme. However, as is discussed later, Bennett [39] points out that reversibility can be simulated on a laboratory computer if the computational history is kept to regenerate the initial state. In this case, the total number of bits in the system, including programme and history bits, are conserved as discussed in detail in Section 4.2. This allows Landauer’s principle to identify the conditions under which the thermodynamic entropy aligns with the algorithmic entropy.
- As there is only one reversible forward path to a particular microstate in the natural world, provided the full details of the microstate are specified, there can be no shorter algorithmic description than that provided by the reversible path. Reversibility also implies that there is maximum mutual information between the initial state and the final state of a computation, as the computational path must pass through the initial state and, as a consequence, Equation (1) will have an equal sign. i.e.,
  $$H\left(s_{i}\right)=H\left(s_{0}\right)+H\left(s_{i}|s_{0}^{*}\right).$$

### 4.1. Why Bits Are Conserved

If an irreversible reference UTM is used to fully capture the reversible process of a real-world computation generating the halt state, $s_{h}$ from the initial state $s_{0}$, reversibility must be maintained.

In the laboratory computer, the programmer, by understanding the behaviour of the computational gates, sets the initial bits to bring about the desired computation. As a consequence, from a laboratory perspective, the full initial state is strictly $\left(p_{h},s_{0}\right)$. From a real-world perspective, $s_{0}$ itself is the programme string, manipulated by the real-world gates. Here $p_{h}$ is needed to specify the instructions embodied in the real-world gates as distinct from the string specifying the actual real-world states. Zurek [40] argues that reversibility in the laboratory UTM can only be achieved by filling up the computer memory with historical records [24,26,39]. In this case, the observed halt state, denoted by ${\hat{s}}_{h}$, ignores the history of the computation. The history is denoted by $g(s_{0}^{*},s_{h})$, or $g_{h}$ for short [40]. The complete final state, including the history, is $s_{h}=\left({\hat{s}}_{h},g_{h}\right)$. From a laboratory perspective, as the computation progresses, programme bits either become the bits to define ${\hat{s}}_{h}$, or remain to retain reversibility. Once programme bits are tracked separately from state bits, $g_{h}=p_{h}=p_{h}^{-1}$, as $p_{h}$ is reversible.

While the programme $U_{W}\left(s_{0}\right)={\hat{s}}_{h},g_{h}$, where *W* is the reversible real-world UTM with its real-world gates, in the laboratory, the computer UTM denoted by *U* implements the programme $p_{h}$ that represents the bit settings that generate $s_{h}$ given $s_{0}$ [36,39]:

$$U\left(p_{h},s_{0}\right)={\hat{s}}_{h},g_{h},$$

and, because of reversibility,

$$U\left(g_{h},{\hat{s}}_{h}\right)=p_{h},s_{0}.$$

As the trajectory through bit space reversibly generates the final state together with the history bits, the algorithmic entropy is conserved provided the history is kept. As a consequence,

(2) $$H\left(p_{h},s_{0}\right)=H\left(s_{h}\right)=H\left({\hat{s}}_{h},g_{h}\right)=H\left(s_{0}\right)+H\left(s_{h}|s_{0}^{*}\right).$$

On the other hand, bits conserved in an isolated thermodynamic system particles such as hydrogen and oxygen may not be. Rather, when the bits specifying position states reduce as particles combine, the bits specifying the momentum states increase to compensate.

### 4.2. Net Entropy Flows and the Algorithmic Entropy

It is shown here that the algorithmic entropy is a function of state, and tracking bit flows yields the same algorithmic entropy as that specified by a halting algorithm.

It is customary to argue that an algorithm that generates all integers in turn on a laboratory computer is of the following form:

$$\begin{array}{cc} N= & 0\\ A.\;\;\;N= & N+1;\;GO:T0\;A \end{array}$$

and is much shorter than one that generates a specific integer and then halts. Eventually, the above algorithm will compute the integer $1,048,576=2^{20}$ and continue without halting. It is often argued that the programme that halts when specifically outputting $2^{20}$ needs extra instructions of a length of 20 bits ($=log_{2}2^{20}$) to specify when to halt. This halting programme must therefore be 20 bits longer than the simple programme above. While this makes sense for a laboratory computation, the argument ignores the actual details of what is required in a real-world computation. Firstly, replacing *N* by N+1 is not reversible and cannot represent a real-world computation, as information is thrown away each cycle. Secondly, a bit is not a “0” or a “1” on some printout but requires energy to set the bits that exist in the physical states of atoms and molecules. Once the energy requirements to store a bit are taken into account, the number of required bits grows as $log_{2}N$. The energy to create these bits must come from somewhere. While $log_{2}N$ grows slowly, ultimately, for such an ongoing computation, there would be insufficient stored energy in the universe to specify the latest integer. If bits are tracked properly, and provided reversibility is kept, the number of bits to generate the *N*th integer is the same, whether the algorithm halts or continues indefinitely.

Indeed, most real-world computations do not halt, but are ongoing as the system moves from one configuration to another. It is shown below that the number of bits is a function of the halt state and can be established by tracking the bit flows in and out of the system.

Consider a system where the initial state at time zero is $s_{0}$, and which contains $H(s_{0})$ bits. Let p* be the shortest reversible real-world programme that drives the system to the configuration of interest and then halts. The programme p* includes both the second law algorithm driving the system to equilibrium, such as the instructions to ignite hydrogen and oxygen to produce heat and water, as well as the instructions already in the system. Let σ refer to the states of the species entering the system, recognising that the natural laws accompanying these species also become part of the programme p*.

p*, σ, and the initial state $s_{0}$ either make up the computational resources that are either in the system orenter it to generate the microstate of interest denoted by ϵ. In order to maintain the system, the process must eject the waste identified by the string ζ. The real-world computation, which contains the reversibility information corresponds to

$$U\left(p^{*},s_{0},\sigma \right)=\epsilon_{k},\zeta_{l}.$$

If no waste is ejected, no information is lost. The algorithmic entropy of the final microstate state at this instant must include the waste string $\zeta_{l}$ that contains the history bits needed for reversibility. Here, because only algorithmic entropy differences are considered, there is no need for an O(1) constant as the computations are UTM-independent. Furthermore, as the computations are ongoing, there is no need to include bits that link subroutines. With this in mind, the result is

$$H\left(\epsilon_{k},\zeta_{l}\right)=H\left(\sigma \right)+\left|p^{*}\right|+H\left(s_{0}\right).$$

H(ϵ,ζ) cannot be separated into H(ϵ) and H(ζ). However, $H\left(\epsilon_{k},\zeta_{l}\right)=H\left(\epsilon_{k}\right)+H\left(\zeta_{l}|\epsilon_{k}^{*}\right)$. The microstate ϵ can be sustained if $H\left(waste\right)=H\left(\zeta_{l}|\epsilon_{k}^{*}\right)$ bits are ejected as waste. In this case,

$$H\left(\epsilon_{k}\right)=H\left(\epsilon_{k},\zeta_{l}\right)-H\left(waste\right)=H\left(\sigma \right)+\left|p^{*}\right|+H\left(s_{0}\right)-H\left(waste\right).$$

Or,

$$H\left(\epsilon_{k}\right)=H\left(\sigma \right)+\left|p^{*}\right|+H\left(s_{0}\right)-H\left(waste\right).$$

Here H(waste) includes heat bits, programme bits, and bits specifying the position states of species that leave the system. This shows that the number of bits $H(\epsilon_{k})$ in the halt state equals the bits in the initial state, plus the number of bits that have entered the system, less the bits in the waste ejected at the halt instant. This allows heat and algorithmic entropy flows into and out of a system to be related to thermodynamic entropy flows, without the need to identify the shortest algorithm that halts to define the final state. If the system resides in a homeostatic set of states, all having the same algorithmic entropy $H(\epsilon_{k})$, the input per unit time of the stored energy in the resource string σ must equal the heat ejected by the system over the same time period, to prevent any energy build up in the system. The greater H(waste) is for a given input, the shorter the instantaneous algorithmic description is, and, as the system moves further from equilibrium, the more ordered it becomes in the sense that it embeds more stored energy. Interestingly, Schneider and Kay [41] have measured the energy dissipation for an ecology and have shown that the more biologically complex the ecology, the more effectively it processes inputs and, as a consequence, the more comprehensively stored energy is transformed into heat.

## 5. Application to the Second Law of Thermodynamics

### 5.1. Non-Equilibrium States and Fluctuations within Equilibrium

If a system exists in an improbable configuration outside the most probable equilibrium set of microstates, the thermodynamic entropy is said to increase as the system trends to equilibrium. However, as the thermodynamic entropy is a property of a macrostate, it does not have an agreed meaning for a fluctuation away from the most probable set of states [42]. On the other hand, because the algorithmic entropy is a property of each microstate, it provides a consistent understanding of what happens as the fluctuation trends to equilibrium. The algorithmic approach recognises that the characteristic of a typical or equilibrium microstate is that most of the energy is associated with momentum degrees of freedom rather than with stored energy states. The trend to a typical or equilibrium state is the trend for stored energy to become heat, increasing the system’s temperature. However, the critical point is that, in an isolated reversible system, all microstates in the same macrostate have the same algorithmic entropy. A fluctuation away from a typical or highly probable state shifts the system to a less probable one, but the algorithmic entropy does not change because the fluctuation follows a reversible path. What has changed is that bits that previously defined momentum degrees of freedom now become bits that define stored energy degrees of freedom. This is consistent with the argument that the von Neumann entropy of a non-equilibrium isolated system, which is described by a time-dependent Hamiltonian, is invariant over time [43]. For example, when the molecules in a system of hydrogen and oxygen ignite, the system trends toward equilibrium, as bits specifying stored energy states become bits specifying momentum states. However, depending on the temperature, once equilibrium is reached, the most probable set of microstates of the system will consist mainly of water as steam, with only traces of hydrogen and oxygen. There is a finite, but low probability of returning to the initial state that consists mainly of hydrogen and oxygen, as rare collision processes can, in principle, split the bonds in water molecules creating separate hydrogen and oxygen molecules.

The shift of bits from momentum degrees of freedom to stored energy degrees of freedom can be understood by defining the string $s_{i}=e_{i},p_{i}$, where $e_{i}$ represents the stored energy contribution, and $p_{i}$ represents the momentum contribution to the specification of $s_{i}$. However, as the stored energy bits and the momentum bits are not additive, the overall algorithmic entropy of a particular microstate in an isolated system is given by

(3) $$H\left(s_{i}\right)\leq H\left(e_{i}\right)+H\left(p_{i}\right).$$

Nevertheless, a simple relationship between these three strings can be derived if the substrings $e_{i}$ and $p_{i}$ are assigned to a separate subset defined by *r*, where *r* denotes the fraction of energy in the momentum degrees of freedom for this subset. As each *r* categorises a unique subset of all the states, the probability of choosing a string at random belonging to subset *r* is denoted by $P_{r}$. For a typical state in the overwhelmingly probable equilibrium set, $P_{r}$ is relatively close to 1. However, it is low for a non-typical fluctuation, where energy is transferred to the stored energy states. In essence, the difference between a highly ordered fluctuation and a typical string in the macrostate is that the extremely large $-log_{2}\left(probability\right)$ term pads out the contribution of $e_{i}$ and $p_{i}$ to the algorithmic entropy of a string in class *r*. Within the class, the stored energy contribution, the momentum contribution and the probability contribution are additive, not sub-additive. As a consequence, the algorithmic entropies of all members of the macrostate are the same. The following details the argument.

Let $n_{e}^{\left(r\right)}$ be the number of stored energy substrings, and $n_{p}^{\left(r\right)}$ be the number of momentum substrings in the subset *r*. The total number of strings with the fixed energy in *r* is $n_{e}^{\left(r\right)}n_{p}^{\left(r\right)}$. All combined strings in the subset are equally likely, and the probability of selecting a particular string is $1/(n_{e}^{\left(r\right)}n_{p}^{\left(r\right)}).$ If $P_{r}\left(i\right)$ is taken to be the overall probability of selecting string $s_{i}$ within *r* out of all possible microstates, then $P_{r}\left(i\right)=P_{r}/\left(n_{e}^{\left(r\right)}n_{p}^{\left(r\right)}\right)$. Shannon’s noiseless coding theorem shows that the algorithmic entropy denoted as $H(s_{i}^{\left(r\right)})$, of a string $s_{i}^{\left(r\right)}$ from the set *r*, is within one bit of $-log_{2}P_{r}\left(i\right)$ [35]. Hence, Inequality (3) can be replaced by the more useful equation, which shows that the algorithmic entropy of the electronic and momentum states are separable for each probability class:

$$H\left(s_{i}^{\left(r\right)}\right)=-log_{2}P_{r}\left(i\right)=-log_{2}P_{r}+log_{2}n_{e}^{\left(r\right)}+log_{2}n_{p}^{\left(r\right)},$$

or

(4) $$H\left(s_{i}^{\left(r\right)}\right)=H\left(P_{r}\right)+H\left(n_{e}^{\left(r\right)}\right)+H\left(n_{p}^{\left(r\right)}\right).$$

As all microstates have the same algorithmic entropy,

$$H\left(s_{i}^{\left(r\right)}\right)=H\left(s_{typical}\right).$$

Furthermore, $H(P_{typical})$ is negligible compared with the bits needed to specify $e_{\left(typical\right)},p_{\left(typical\right)}$. For such a typical or equilibrium state, the algorithmic entropy can be taken to be

(5) $$H\left(s_{typical}\right)=H\left(n_{e}\left(typical\right)\right)+H\left(n_{p}\left(typical\right)\right).$$

In the set of all possible configurations, the typical configuration is random relative to a low probability configuration. As $H(P_{r})$ is the number of extra bits that need to be added to the specification of $s_{i}\left(r\right)=e_{i}^{\left(r\right)}p_{i}^{\left(r\right)}$, to create a typical configuration, it is a measure of the distance that the configuration of interest is from an equilibrium or random one. Hence, we can define $D(s_{i}^{\left(r\right)})$ as the difference between a non-equilibrium state and a typical one as

$$D\left(s_{i}^{\left(r\right)}\right)=H\left(P_{r}\right).$$

When the fluctuation with probability $P_{r}$ reverts to equilibrium, $H(P_{r})$ bits transfer to the most probable set of states, increasing the thermodynamic entropy by $k_{B}ln2H\left(P_{r}\right)$.

The process can be illustrated with a simple example. Consider a string of length 1000, which represents a microstate of an isolated thermodynamic system. There are $2^{1000}$ similar strings. Assume a requirement, such as the conservation of energy, where 80% of the binary characters specifies that the string is 1, and 20% specifies it is 0. The Shannon entropy of the set of these strings is then 722, and there are $2^{722}$ potential strings satisfying the requirement. From an algorithmic perspective, each can be coded by 722 bits.

The most probable set of strings are those showing no pattern, corresponding to a random arrangement of the 800 ones and 200 zeros. However, the simplest string is 800 ones followed by 200 zeros, i.e., $1^{800}0^{200}$ and its converse $0^{200}1^{800}$. The first section of the 800:200 string can be coded by $log_{2}\left(800\right)=10$ bits, while the 200 zero section can coded by 8 bits. This highly ordered 800:200 string and its converse can be coded with a little more than 18 bits compared with 722 bits for the typical string. Here extra overhead bits and the requirements of self-delimiting coding are ignored to simplify the discussion. The probability of the 800:200 string and its converse in the set of all strings is 2 in $2^{722}$. Equation (4) shows that the algorithmic entropy of this particular string in the set of all such strings will be $-log_{2}\left(P_{r}\right)=721+log_{2}2$, as there are only two strings in the subset *r*. However, the probability for a typical string is H(typical)≈722. Thus, the 721 bits needed to specify the probability of the highly ordered state, when transferred to the momentum states, shift the initial ordered string to an equilibrium one.

If an improbable microstate is created in a system, it will trend to equilibrium. However, if the system is to be maintained in a non-equilibrium state belonging to subset *r*, $D(s_{i}^{\left(r\right)})$ bits, corresponding to the increase in thermodynamic entropy, must be ejected from the system mainly as heat, and the same number of bits must enter the system as stored energy, for the system. An example might be a laser where excited atoms are maintained by the input of light from a discharge tube, or a system of algae sustained from death and decay by accessing photons from the sun to replicate, while ejecting decayed products as high entropy waste. Once the input source is no longer accessible, the system reverts to its decayed equilibrium state where stored energy has become thermal energy. What would seem as just a fluctuation in an isolated system can be sustained in a homeostatic stable configuration provided stored energy enters, and high entropy waste is ejected to ensure that the number of bits $D(s_{i}^{\left(r\right)})$ is maintained in the far-from-equilibrium subset *r*.

### 5.2. The Trajectory Approach

Zurek in an appendix [35] argues that the physical laws driving a system from an ordered state $s_{0}$ to equilibrium can be can be understood from an AIT framework as the computational trajectory that shifts $s_{0}$ to a more random halt state $s_{h}$, after $t_{h}$ computational steps. The Zurek approach deals with the situation where the initial configuration belongs to a restricted macrostate that is not a fluctuation from the equilibrium set of states. In order for the system to reach equilibrium, bits must be added for the state spaced to expand. The approach can be illustrated by considering a real-world system of N magnetic spins where a “1” is used to denote a spin up and a “0” a spin down. Let the initial ordered state be one where all the spins are aligned up. This is a frozen state, as it requires the temperature of the system to be lower than the Curie temperature. The system string in the frozen state can be represented by $1^{N}y_{1}y_{2}\ldots y_{N}$. Here, $y_{1}y_{2}\ldots y_{N}$ will be denoted by $Y_{i}\left(T\right)$, where $Y_{i}\left(T\right)$ denotes the different variations of the momentum states for a system belonging to the macrostate at temperature *T*. The algorithmic entropy as $H=log_{2}N+H\left(Y_{i}\left(T\right)\right)$. Here, $H(Y_{i}\left(T\right))$ is the number of bits needed to specify each momentum state. The computation that generates the trajectory of the system from the initial states $s_{0}$ to the halt state $s_{h}$ in $t_{h}$ steps is the routine

$$U^{t_{h}}s_{0}=s_{h}.$$

*U* captures the deterministic law of motion that shift one discrete state to another, simulating the gates embodying natural laws. Hence,

$$H\left(s_{h}\right)=H\left(t_{h}\right)+H\left(U\right)+H\left(s_{0}\right).$$

Eventually, the length of the algorithm is dominated by $t_{h}$. As $t_{h}$ increases for each new halt state, the algorithmic entropy increases. This increases from the laboratory perspective is seen as the programme bits defining $t_{h}$ entering the system, expanding the state space, and in so doing, driving the configuration trends toward an equilibrium state. The overwhelming majority of states occur in the region when $t_{h}\approx \Omega /2$, where Ω is the number of possible states. In this case, $log_{2}t_{h}≃log_{2}\Omega$ corresponding to the Shannon entropy of the available states [34].

However, from the perspective of the spin example above, the system is initially frozen in the highly ordered state. This initial ordered state is not a typical state in the full system but belongs to a smaller subset specified by a restricted macrostate. If the whole system is to trend toward the ultimate equilibrium, the macrostate must expand as indicated by bits entering the system to increase the temperature. The increase in disorder given by the increase in $log_{2}t_{h}$ for each successive halt state, captures the process where heat from the larger surrounding system, corresponding to programme bits, enters the subsystem. However, once equilibrium is reached, a fluctuation from equilibrium could re-align the magnetic spins. However, in this case, the fluctuation does not reproduce the original ordered state as bits would need to be removed from the system.

In this situation, the distance the initial state $s_{0}$ is from a typical equilibrium configuration $s_{equil}$ is the number of bits that must enter the system to generate $s_{equil}$. This is given by

$$D\left(s_{0}\right)=H\left(s_{equil}\right)-H\left(s_{0}\right).$$

This measure is analogous to Kolmogorov’s deficiency in randomness.

## 6. Landauer’s Principle

### 6.1. Tracking Bit Flows between Stored Energy Degrees of Freedom and Momentum Degrees of Freedom

Before discussing Landauer’s principle, the relationship between the bits specifying stored energy states and momentum states in an isolated system needs to be explored. This can be illustrated with a representative or archetypical system of an isolated gas of *N* atoms. In terms of particle interactions, the atoms behave as an ideal gas, but each atom has a ground state and an excited state that stores ϵ Joules.

Let the initial state of the system be one where the temperature is low, but where all the electronic states have been excited by a flash lamp or laser before completely isolating the system. The initial thermodynamic entropy is considered to be low, but, from an algorithmic point of view, bits are conserved. As the second law of thermodynamics disperses the stored energy, the number of bits needed to specify the electronic states falls, but there is a corresponding increase in bits needed to specify the momentum states. The temperature rises as the number of available momentum states increases until the system settles in the equilibrium set of states.

Over time, a fluctuation can repopulate some of the electronic states, but the algorithmic entropy of this fluctuation is the same as that for a typical equilibrium microstate.

The number of bits needed to specify the microstate $s_{i}$ in the isolated system is determined by $\Omega =2^{N}$. The number of ways the total energy *E* of the macrostate can be distributed between the electronic and momentum states. Each electronic state can store ϵ Joules if the state is occupied; otherwise, the energy stored is zero. Because energy is conserved, not all states are allowed and, as in Equation (3), the algorithmic entropy is sub-additive. Instead, the relationship between the algorithmic entropy of the stored energy and the momentum states is given by Equation (4).

As up to Nϵ units of energy can reside in the *N* electronic states, the energy units assigned to the momentum states must lie between E−Nϵ and *E*. Because the different mix of electronic and momentum states all belong to the same macrostate with energy *E*, each microstate can be specified by $N=log_{2}\Omega$ bits.

If the system is at temperature *T*, some electronic states will be occupied. In this case, the number of bits in an occupied electronic states is $\epsilon /(k_{B}ln2T)$. As is discussed later in the next section, these bits must be erased from the electronic state to reversibly transfer ϵ Joules to the momentum states.

### 6.2. The Principle

This section explores how Landauer’s principle [26] can relate the bit flows of algorithmic entropy to thermodynamic entropy changes. Landauer’s principle arose out of the paradox of Maxwell’s demon. Prior to Landauer’s insight, Szilard [44] and later Brillouin [45], in attempting to save the second law of thermodynamics from Maxwell’s demon, argued that acquiring information involved an entropy cost of at least $k_{B}ln2$ per bit. Landauer showed that it was not the entropy or energy cost of acquiring bit that was critical; rather, the demon was foiled by having to pay $k_{B}ln2T$ Joules for every bit that it needed to erase from a system at temperature *T*. It is only the reversible erasure of a bit that corresponds to $k_{B}ln2T$ Joules, as a bit is physical and the erasure of a bit in the system by a natural process must create a corresponding bit in the environment. Irreversible erasure costs more, increasing the total thermodynamic entropy of the universe. As Lloyd [46] puts it, ‘Essentially, the one-to-one dynamics of Hamiltonian systems implies that when a bit is erased the information that it contains has to go somewhere. If the information goes into observable degrees of freedom of the computer, such as another bit, then it has not been erased but merely moved; but if it goes into unobservable degrees of freedom such as the microscopic motion of molecules it results in an increase of entropy of at least $k_{B}ln2$’. From a physical point of view in the natural world, erasure corresponds to a decrease in the number of states accessible to the system. If, for example, heat is removed from a system maintained at constant pressure by a piston, the volume must contract, as the number of position bits decreases. When the system is no longer reversible, the cost of removing a bit is greater than $k_{B}ln2T$ Joules of heat.

However, before exploring Landauer’s approach, we need to be clear what, in this context, a bit is. While a “0” or a “1” in a computer memory is often represented by the level of charge in a capacitive memory unit, a bit is distinct from its method of storage. Again, one might specify the ground state of an atom by the character “0” and the excited state by a “1” separated by ϵ Joules. However, these characters do not represent bits in the Shannon or computational sense. Landauer’s argument used a symmetrical bi-stable well as the storage device where both the “0” and the “1” carry the same energy, but are separated by a potential barrier. He used the term “RESTORE TO ONE” to denote the process that manipulates the potential barrier and the relative depth of the wells to ensure the “ZERO” well empties at the temperature *T*. Whichever side of the bi-stable well was originally occupied, the process of resetting dissipates at least $k_{n}ln2T$ Joules for a system at temperature *T*. The energy associated with a bit in the context of Landauer’s principle refers to the energy required to move a bit relative to the background thermal noise. In a reversible system, the energy dissipated increases the number of bits in the momentum degrees of freedom.

However, readers should be aware that, while Landauer’s principle is widely accepted, there is ongoing discussion on the underlying physics and philosophical issues, and there is criticism of its applicability [47]. Some concerns surround the operation of functioning computers and whether logical reversibility implies thermodynamic reversibility and vice versa [48]. Norton has introduced a ‘no-go’ principle’ that sees fluctuations in a system as undermining reversibility, while del Rio et al. [49] have considered the effect of quantum entanglement in the observer’s memory. Ladyman and Robertson review some key issues [50] and the ‘no-go’ principle’.

Irrespective of the philosophical issues, recent experimental evidence supporting Landauer’s principle is strong [51,52,53,54].

However, in the approach here, the system is considered to be classical, fluctuations are deterministic and not stochastic, and the “no-go” argument is irrelevant, as are arguments about the practicalities that might arise in constructing reversible computers. Here, Landauer’s principle follows directly from the conservation of bits in an isolated system as discussed in Section 4.1. Consider an isolated system consisting of two subsystems. One of these is the subsystem of interest, in contact with the other, which is the surrounding environment at temperature *T*. Reversible removal of one bit from the subsystem of interest adds one bit to the environmental subsystem corresponding to a transfer of $k_{b}ln2T$ Joules. As has been emphasised, if reversibility is lost, the energy cost is greater.

The temperature dependence of the energy associated with a bit can be understood in terms of Shannon’s so-called ‘ultimate limit’. This limit identifies the minimum average energy of $ln2N_{0}$ Joules to send one bit through a communication channel at the channel capacity. $N_{0}$ is the noise spectral power density. As the noise power density can be taken to be $k_{B}T$ in a system at temperature *T*, for a bit to be recognised against the background noise, the energy per bit must be $\geq k_{B}ln2T$ Joules. This is consistent with Landauer’s principle suggesting that a real-world computer operates at the ultimate limit. The Shannon limit indicates why more energy must be associated with each bit when the temperature rises as the bit needs to be identified above the thermal background. Even though the real-world does not know what temperature is, when physical laws drive the system from one state to another, for the observer, “bit language” and temperature are useful constructs.

The concept can be illustrated by a two-dimensional matrix of N randomly aligned magnetic spins with $2^{N}$ possible microstates. Because N is the Shannon entropy of all states, the unit of measure is clearly a bit, and not just an arbitrary binary representation. The spin-up and the spin-down states have the same energy. In this case, the erasure operation ‘RESTORE TO ONE’ can be achieved by applying a slight magnetic field to lower the potential energy of the spin-up state corresponding to “ONE”. The dissipated energy goes to the momentum degrees of freedom. If the system temperature is below the Curie temperature, once a few spins align, coupling between spins can align all spins, in effect setting all spins to “ONE”. In this case, the number of magnetic states reduces from $2^{N}$ to 1. Strictly, the alignment of all spins to “0” rather than “1” is possible, but this is energetically unlikely once the “1” spin direction is frozen. Because $log_{2}1=0$, the corresponding statistical entropy becomes 0. The bits in the magnetic system decrease, but bits increase in the momentum system when disorder as latent heat is transferred to the momentum degrees of freedom. Provided the detailed computational path is kept, the information to provide reversibility still resides in the system, and there is no change in the system algorithmic entropy and hence the thermodynamic entropy. If $k_{B}ln2T$ Joules are required to erase one bit, $k_{B}ln2T$ Joules can restore the system to the original microstate only if the reversible path is exactly followed. However, as Landauer showed, once heat has passed from the momentum subsystem to an external sink, degrees of freedom within the system are lost and all the information on the prior microstate has been erased from the system. At that point, the system’s state space is compressed as bits have been erased but the environmental state space expands. The entropy cost of restoring the system increases in thermodynamic terms.

#### An Additional Perspective of the Algorithmic Approach

While the algorithmic approach effectively provides the same explanation for erasure as Landauer, there is a slight but important difference. From Landauer’s perspective, as there is only one microstate after spin alignment, and because $log_{2}1=0$, the statistical entropy of the spin system drops from N to zero. On the other hand, the algorithmic approach assigns a residual algorithmic entropy of $log_{2}N$ bits to the string after alignment, as this is the number of bits required to specify, or code, the N identical characters. It is only when the magnetic atoms are replaced by non-magnetic atoms will this contribution to the algorithmic entropy actually be zero. This is consistent, as there is now a clear distinction between an aligned magnetic system and a non-magnetic system.

### 6.3. Landauer’s Principle and Resource Use of Natural Systems

Tracking bits specifying the algorithmic entropy is completely consistent with the second law and the thermodynamic entropy. Where bits are conserved, bit flows within the system can be tracked independently of the temperature, as reversibility is maintained. However, for inflows and outflows of heat denoted by dQ at a temperature *T*, the thermodynamic entropy change is S=dQ/T. This corresponds to a transfer of N bits, where $N=dQ/(k_{B}ln2T)$. For example, a transfer of $k_{B}ln2$ bits from a system at temperature $T_{0}$ to the environment at a lower temperature $T_{1}$ corresponds to a heat transfer of $k_{B}ln2T_{0}$ Joules. If reversibility is kept at the microscopic level, the bit can be transferred back at exactly $k_{B}ln2T_{0}$ Joules. However, once the reversible path information disappears, i.e., correlations between the particles in the environment and the system is lost, as $T_{1}<T_{0}$, the thermodynamic entropy in the environment, increases to $k_{B}ln2T_{0}/T_{1}$. In different words, $k_{B}kn2$ bits are erased from the system by interaction with the environment, and $k_{B}ln2T_{0}/T_{1}$ bits appear in the environment, expanding the momentum degrees of freedom as required by the second law of thermodynamics. This increase in bits in the environment comes from the programme bits that were needed to maintain reversibility, and which did not initially contribute to the thermodynamic entropy. Once the energy becomes dispersed, they become momentum bits contributing to the thermodynamic entropy.

In addition to the stored energy bits, and those specifying momentum degrees of freedom, in a real-world system, when molecular species enter or leave the system, the position degrees of freedom expand or contract. Species that enter or leave a system may transfer heat bits, stored energy bits, and bits representing position degrees of freedom.

When bits as stored energy enter the system, they do not initially contribute to the thermodynamic entropy. From the algorithmic perspective, the number of bits in the system increases, and, if residual stored energy bits leave, the algorithmic entropy reduces. However, it is only as the stored energy diffuses through the system, driving the system to the most probable set of states, does the thermodynamic entropy increase, as the number of momentum bits increases. It is not that one bit of information is carried by $k_{B}ln2T_{0}$ Joules. Rather, it takes at least $k_{B}ln2T_{0}$ Joules to erase one computational bit from a system. Only when the process is reversible can the erased bit be restored with $k_{B}ln2T$ Joules.

### 6.4. Interpreting Bit Transfers in a Far-From-Equilibrium System

Section 6.1 outlined the simple archetypical model of a non-interacting gas, where each atom with a ground state and excited state. This can be used to understand how a general, far-from-equilibrium, thermodynamic system is maintained.

Imagine such a gas system bathed in radiation that continually excites the electronic states while the electronic states decay, passing heat to the momentum degrees of freedom under second law processes. This simple model captures some of the features of a far-from-equilibrium system, such as the biosphere where energy is captured from the sun. If at an instant the system is isolated, bits are conserved as the microstate trends toward the most probable set of states. The bits originally embodied in stored energy as “potential entropy” become bits in the momentum degrees of freedom to become “realised entropy”. However, if the flow of bits is maintained, and the heat and waste is removed at an appropriate rate, the system can settle in a homeostatic stable state where the energy captured by the electronic states equals the heat ejected and the input of the entropy in bits equals the outflow. At any instant, in the homeostatic state, not all electronic states will be occupied as energy is continually transferring to the momentum states. However, when the temperature becomes stable at $T_{0}$, if there are $N_{e}$ electronic states, the energy captured in the electronic states corresponds to $N_{e}k_{B}ln2T_{0}$ Joules and the heat removed from the system to maintain homeostasis also corresponds to $N_{e}k_{B}ln2T_{0}$ Joules. In other words, $k_{B}ln2T_{0}$ Joules are needed to move one bit (i.e., erase it from one subsystem and move it to another) against the thermal background.

Devine [22,23,27] uses the above argument to establish the entropy cost of maintaining stability in a far-from-equilibrium system at $T_{0}$. The system is maintained by accessing stored energy and ejecting waste. For example, plants capture the energy from the sun, driving the system further from equilibrium than if no life existed. However, plants continually expel waste and degrade. In order to be maintained, both bits and energy in must equal the bits and energy out. The net transfer of position degrees of freedom must also be zero as species enter and degraded species leave. A bacterial system can be maintained at its carrying capacity as replication processes use energy inputs to continually re-create bacterial structures at a rate that counter the inevitable death and decay due to the second law of thermodynamics [22].

However, if the output temperature in a natural, far-from-equilibrium system rises to $T_{1}$, as might happen if less heat can be extracted, $N_{e}k_{B}ln2T_{1}$ Joules are needed to eject $N_{e}$ bits and the input temperature will need to rise to ensure the input of energy and bits coincides with the energy and bits ejected by the system. Nevertheless, the system will become more disordered as is happening to the biosystem with global warming. Insufficient entropy is being properly ejected.

## 7. Conclusions

This paper provides a conceptual framework that identifies how the computational behaviour of a real-world system determines its thermodynamic entropy providing a framework to analyse stable, non-equilibrium systems. The approach differs from traditional thermodynamics in three ways.

Firstly, the algorithmic entropy, like energy, is a measure for each microstate in a given macrostate, whereas the other entropy measures are a property of the macrostate itself, or the system as a whole.

Secondly, the algorithmic entropy for all microstates in an isolated system are the same, irrespective of whether the microstate belongs to the most probable or equilibrium set, or whether it is a fluctuation away from this set. The argument of Section 3 shows that, in this situation, the algorithmic entropy of a microstate is the same as the thermodynamic entropy of the macrostate allowing for the conversion factor of $k_{B}ln2$ to turn bits to entropy.

Thirdly, fluctuations within an isolated system are not strictly more ordered from an algorithmic perspective. Rather, a fluctuation represents a shift in bits specifying the momentum states to bits specifying the stored energy states, but with no change in algorithmic entropy. As a fluctuation is not stable, the system will trend toward the most probable or equilibrium set of microstates when the bits specifying stored energy states shift back to specify momentum states. The bits in the stored energy states of a configuration that is distant from the most probable set of states is, from the algorithmic perspective, “potential thermodynamic entropy”. Once the stored energy is dispersed through the system, these bits become the “realised thermodynamic entropy” corresponding to $k_{B}ln2$ per bit.

It is shown that, as the algorithmic entropy is a function of state, the algorithmic entropy determined by the halt requirement has the same number of bits as that determined by tracking the bit flows giving rise to the same particular microstate. When fully accounted for, bits are conserved, as a bit is physical. As from Landauer’s principle, the cost of reversibly erasing one bit from a thermodynamic system at temperature *T* is $k_{B}ln2T$ Joules, the corresponding thermodynamic entropy change is then $k_{B}ln2$. Irreversible erasure increases the entropy in the environment by an even greater amount, because the programme bits that capture reversibility are erased to become bits specifying environmental momentum states.

The distance a particular microstate is from an equilibrium or typical microstate, whether it is an ordered microstate or a fluctuation from equilibrium, is the number of bits that must move from the stored energy microstate in order to specify a typical equilibrium state.

## Abbreviations

The following abbreviations are used in this manuscript:

AIT	algorithmic information theory
UTM	universal Turing machine
AMSS	algorithmic minimum sufficient statistic

## References

[1] Martin-Löf P. (1966). The definition of random sequences. Inf. Control.

[2] Gács P. (1980). Exact Expressions for some randomness tests. Zeitschr. f. Math. Logik und Grundlagen d. Math..

[3] Chaitin G. (1974). Information-theoretic Computational Complexity. IEEE Trans. Inf. Theory.

[4] Vitányi P.M.B., Li M. (2000). Minimum description length induction, Bayesianism, and Kolmogorov complexity. IEEE Trans. Inf. Theory.

[5] Li M., Chen X., Li X., Ma B., Vitanyi P. (2004). The similarity metric. IEEE Trans. Inf. Theory.

[6] Ferragina P., Giancarlo R., Greco V., Manzini G., Valiente G. (2007). Compression-based classification of biological sequences and structures via the Universal Similarity Metric: Experimental assessment. BMC Bioinform..

[7] Hutter M. (2007). On universal prediction and Bayesian confirmation. Theor. Comput. Sci..

[8] Chaitin G., Hogrebe W., Bromand J. (2004). On the intelligibility of the universe and the notions of simplicity, complexity and irreducibility. Grenzen und Grenzüberschreitungen, XIX. Deutscher Kongress für Philosophie, Bonn, September 2002.

[9] Calude C.S., Meyerstein F.W. (1999). Is the universe lawful?. Chaos Solitons Fractals.

[10] Hutter M. (2010). A Complete Theory of Everything (Will Be Subjective). Algorithms.

[11] Davies P.C.W. (2003). The Fifth Miracle: The Search for the Origin of Life.

[12] Li M., Vitányi P.M.B. (2008). An Introduction to Kolmogorov Complexity and Its Applications.

[13] Zenil H., Badillo L., Hernández-Orozco S., Hernández-Quiroz F. (2018). Coding-theorem like behaviour and emergence of the universal distribution from resource-bounded algorithmic probability. Int. J. Parallel Emerg. Distrib. Syst..

[14] Zenil H., Marshall J.A.R., Jesper Tegnér J. (2015). Approximations of Algorithmic and Structural Complexity Validate Cognitive-behavioural Experimental Results. arXiv.

[15] Gauvrit N., Zenil H., Soler-Toscano F., Delahaye J.P., Brugger P. (2017). Human behavioral complexity peaks at age 25. PLoS Comput. Biol..

[16] Zenil H., Kiani N., Tegnér J. (2018). An Algorithmic Refinement of Maxent Induces a Thermodynamic-like Behaviour in the Reprogrammability of Generative Mechanisms. arXiv.

[17] Zenil H., Kiani N.A., Marabita F., Deng Y., Elias S., Schmidt A., Ball G., Tegnér J. (2018). An Algorithmic Information Calculus for Causal Discovery and Reprogramming Systems. BioArxiv.

[18] Zenil H., Gershenson C., Marshall J.A.R., Rosenblueth D.A. (2012). Life as Thermodynamic Evidence of Algorithmic Structure in Natural Environments. Entropy.

[19] Hernández-Orozco S., Kiani N.A., Zenil H. (2018). Algorithmically probable mutations reproduce aspects of evolution, such as convergence rate, genetic memory and modularity. R. Soc. Open Sci..

[20] Devine S. (2014). An Algorithmic Information Theory Challenge to Intelligent Design. Zygon.

[21] Dembski W.A. (2002). Intelligent Design as a Theory of Information. http://arn.org/docs/dembski/wd_idtheory.htm.

[22] Devine S.D. (2016). Understanding how replication processes can maintain systems away from equilibrium using Algorithmic Information Theory. Biosystems.

[23] Devine S. (2018). An economy viewed as a far-from-equilibrium system from the perspective of algorithmic information theory. Entropy.

[24] Bennett C.H. (1982). Thermodynamics of computation—A review. Int. J. Theor. Phys..

[25] Vereshchagin N.K., Vitányi P.M.B. (2004). Kolmogorov’s Structure Functions and Model Selection. IEEE Trans. Inf. Theory.

[26] Landauer R. (1961). Irreversibility and heat generation in the computing process. IBM J. Res. Dev..

[27] Devine S. (2017). The information requirements of complex biological and economic systems with algorithmic information theory. Int. J. Des. Nat. Ecodyn..

[28] Solomonoff R.J. (1964). A formal theory of inductive inference; part 1 and part 2. Inf. Control.

[29] Kolmogorov K. (1965). Three approaches to the quantitative definition of information. Problems Inform. Transmission.

[30] Chaitin G. (1966). On the length of programs for computing finite binary sequences. J. ACM.

[31] Chaitin G. (1975). A theory of program size formally identical to information theory. J. ACM.

[32] Zvonkin A., Levin L. (1970). The complexity of finite objects and the development of the concepts of information and randomness by means of the theory of algorithms. Russ. Math. Surv..

[33] Gács P. (1974). On the symmetry of algorithmic information. Sov. Math. Dokl..

[34] Devine S.D. (2009). The insights of algorithmic entropy. Entropy.

[35] Zurek W.H. (1989). Algorithmic randomness and physical entropy. Phys. Rev. A.

[36] Bennett C.H., Herken R. (1988). Logical Depth and Physical Complexity. The Universal Turing Machine—A Half-Century Survey.

[37] Gács P. (2004). The Boltzmann Entropy and Random Tests. http://www.cs.bu.edfaculty/gacs/papers/ent-paper.pdf.

[38] Jaynes E.T. (1965). Gibbs vs Boltzmann entropies. Am. J. Phys..

[39] Bennett C.H. (1973). Logical reversibility of computation. IBM J. Res. Dev..

[40] Zurek W.H. (1989). Thermodynamics of of computation, algorithmic complexity and the information metric. Nature.

[41] Schneider E.D., Kay J.J. (1994). Life as a manifestation of the second law of thermodynamics. Math. Comput. Model..

[42] Esposito M., Van den Broeck C. (2011). Second law and Landauer principle far from equilibrium. Europhys. Lett..

[43] Parrondo J.M.R., Horowitz J.M., Sagawa T. (2015). Thermodynamics of information. Nat. Phys..

[44] Szilard S. (1929). Uber die Entropieverminderung in einnem thermodynamischen System bei Eingriffen intelligenter Wesen. Zeitschrift für Physik.

[45] Brillouin L. (1951). Maxwell’s Demon Cannot Operate: Information and Entropy. I. J. Appl. Phys..

[46] Lloyd S. (2000). Ultimate physical limits to computation. Nature.

[47] Rex A. (2017). Maxwell’s demon—A historical review. Entropy.

[48] Kish L.B., Khatri S.P., Granqvist C.G., Smulko J.M. Critical remarks on Landauer’s principle of erasure-dissipation: Including notes on Maxwell demons and Szilard engines. Proceedings of the 2015 International Conference on Noise and Fluctuations (ICNF).

[49] del Rio L., Aberg J., Renner R., Dahlsten O., Vlatko Vedra V. (2011). The thermodynamic meaning of negative entropy. Nature.

[50] Ladyman J., Robertson K. (2014). Going round in circles: Landauer vs. Norton on the thermodynamics of computation. Entropy.

[51] Bérut A., Petrosyan A., Ciliberto S. (2015). Information and thermodynamics: Experimental verification of Landauer’s Erasure principle. J. Stat. Mech. Theory Exp..

[52] Jun Y., Gavrilov M.C.V., Bechhoefer J. (2014). High-precision test of Landauer’s principle in a feedback trap. Phys. Rev. Lett..

[53] Hong J., Lambson B., Dhuey S., Bokor J. (2016). Experimental test of Landauers principle in single-bit operations on nanomagnetic memory bits. Sci. Adv..

[54] Yan L.L., Xiong T.P., Rehan K., Zhou F., Liang D.F., Chen L., Zhang J.Q., Yang W.L., Ma Z.H., Feng M. (2018). Single-atom demonstration of the quantum Landauer principle. Phys. Rev. Lett..

